# Developments in Image Processing Using Deep Learning and Reinforcement Learning

**DOI:** 10.3390/jimaging9100207

**Published:** 2023-09-30

**Authors:** Jorge Valente, João António, Carlos Mora, Sandra Jardim

**Affiliations:** 1 Techframe-Information Systems, SA, 2785-338 São Domingos de Rana, Portugal; jorge.valente@techframe.pt (J.V.); joao.antonio@techframe.pt (J.A.); 2 Smart Cities Research Center, Polytechnic Institute of Tomar, 2300-313 Tomar, Portugal; carlos.mora@ipt.pt

**Keywords:** artificial intelligence, deep learning, reinforcement learning, image processing

## Abstract

The growth in the volume of data generated, consumed, and stored, which is estimated to exceed 180 zettabytes in 2025, represents a major challenge both for organizations and for society in general. In addition to being larger, datasets are increasingly complex, bringing new theoretical and computational challenges. Alongside this evolution, data science tools have exploded in popularity over the past two decades due to their myriad of applications when dealing with complex data, their high accuracy, flexible customization, and excellent adaptability. When it comes to images, data analysis presents additional challenges because as the quality of an image increases, which is desirable, so does the volume of data to be processed. Although classic machine learning (ML) techniques are still widely used in different research fields and industries, there has been great interest from the scientific community in the development of new artificial intelligence (AI) techniques. The resurgence of neural networks has boosted remarkable advances in areas such as the understanding and processing of images. In this study, we conducted a comprehensive survey regarding advances in AI design and the optimization solutions proposed to deal with image processing challenges. Despite the good results that have been achieved, there are still many challenges to face in this field of study. In this work, we discuss the main and more recent improvements, applications, and developments when targeting image processing applications, and we propose future research directions in this field of constant and fast evolution.

## 1. Introduction

Images constitute one of the most important forms of communication used by society and contain a large amount of important information. The human vision system is usually the first form of contact with media and has the ability to naturally extract important, and sometimes subtle, information, enabling the execution of different tasks, from the simplest, such as identifying objects, to the more complex, such as the creation and integration of knowledge. However, this system is limited to the visible range of the electromagnetic spectrum. On the contrary, computer systems have a more comprehensive coverage capacity, ranging from gamma to radio waves, which makes it possible to process a wide spectrum of images, covering a wide and varied field of applications. On the other hand, the exponential growth in the volume of images created and stored daily makes their analysis and processing a difficult task to implement outside the technological sphere. In this way, image processing through computational systems plays a fundamental role in extracting necessary and relevant information for carrying out different tasks in different contexts and application areas.

Image processing originated in 1964 with the processing of the images of the lunar surface, and in a simple way, we can define the concept of image processing as an area of signal processing dedicated to the development of computational techniques aimed at the analysis, improvement, compression, restoration, and extraction of information from digital images. With a wide range of applications, image processing has been a subject of great interest both from the scientific community and from industry. This interest, combined with the technological evolution of computer systems and the need to have systems with increasingly better levels of performance, both in terms of precision and reliability and in terms of processing speed, has enabled a great evolution of image processing techniques, moving from the use of nonlearning-based methods to the application of machine learning techniques.

Having emerged in the mid-twentieth century, machine learning (ML) is a subset of artificial intelligence (AI), a field of computer science that focuses on designing machines and computational solutions capable of executing, ideally automatically, tasks that include, among others, natural language understanding, speech understanding, and image recognition [1]. When providing new ways to design AI models [2], ML, such as other scientific computing applications, commonly uses linear algebra operations on multidimensional arrays, which are computational data structures for representing vectors, matrices, and tensors of a higher order. ML is a data analysis method that automates the construction of analytical models and computer algorithms, which are used in a large range of data types [1] and are particularly useful for analyzing data and establishing potential patterns to try and predict new information [3]. This suit of techniques has exploded in use and as a topic of research over the past decade, to the point where almost everyone interacts with modern AI models many times every day [4].

AI, in particular ML, has revolutionized many areas of technology. One of the areas where the impact of such techniques is noticeable is image processing. The advancement of algorithms and computational capabilities has driven and enabled the performance of complex tasks in the field of image processing, such as facial recognition, object detection and classification, generation of synthetic images, semantic segmentation, image restoration, and image retrieval. The application of ML techniques in image processing brings a set of benefits that impact different sectors of society. This technology has the potential to optimize processes, improve the accuracy of data analysis, and provide new possibilities in different areas. With ML techniques, it is possible to analyze and interpret images with high precision. The advances that have been made in the use of neural networks have made it possible to identify objects, recognize patterns, and carry out complex analyses on images with a high accuracy rate. Pursuing ever-increasing precision is essential in areas such as medicine, where accurate diagnosis can make a difference in patients’ lives.

By applying ML techniques and models to image processing, it is possible to automate tasks that were previously performed manually. In this context, and as an example, we have quality control processes in production lines, where ML allows for the identification of defects in products quickly and accurately, eliminating the need for human inspection, leading to an increase in process efficiency, as well as to a reduction in errors inherent to the human factor and costs.

The recognized ability of ML models to extract valuable information from images enables advanced analysis in several areas, namely public safety, where facial recognition algorithms can be used to identify individuals, and scientific research, such as the inspection of astronomical images, the classification of tissues or tumor cells, and the detection of patterns in large volumes of data.

With so much new research and proposed approaches being published with high frequency, it is a daunting task to keep up with current trends and new research topics, especially if they occur in a research field one is not familiar with. For this purpose, we propose to explore and review publications discussing the new techniques available and the current challenges and point out some of the possible directions for the future. We believe this research can prove helpful for future researchers and provide a modern vision of this fascinating and vast research subject.

On the other hand, and as far as it was possible to verify from the analysis of works published in recent years, there is a lack of studies that highlight machine learning techniques applied to image processing in different areas. There are several works that focus on reviewing the work that has been developed in a given area, and the one that seems to arouse the most interest is the area of medical imaging [5,6,7,8,9]. Therefore, this paper also contributes to presenting an analysis and discussion of ML techniques in a broad context of application.

This document is divided into sections, subsections, and details. Section 2—Introduction: describes the research methodology used to carry out this review manuscript. Section 3—Technical Background: presents an overview of the AI models most used in image processing. Section 4—Image Processing Developments: describes related work and different state-of-the-art approaches used by researchers to solve modern-day challenges. Section 5—Discussion and Future Directions: presents the main challenges and limitations that still exist in the area covered by this manuscript, pointing out some possible directions for the evolution of the models proposed to date. Finally, Section 6 provides a brief concluding remark with the main conclusions that can be taken from our study.

## 2. Methodology

In order to carry out this review, we considered a vast number of scientific publications in the scope of ML, particularly those involving image processing methods using DL and RL techniques and applied to real-world problems.

### 2.1. Search Process and Sources of Information

In order to guarantee the reliability of the documents, the information sources were validated, having been considered reputable publication journals and university repositories. From the selected sources, we attempted to include research from multiple areas and topics to provide a general and detailed representation of the ways image processing research has developed and can be used. Nevertheless, some areas appear to have developed a greater interest in some of the ML methods previously described. The search process involved using a selection of keywords that are closely related to image processing on popular scientific search engines such as Springer Science Direct, and Core. These search engines were selected since they allowed us to make comparable searches, targeting specific terms and filtering results by research area. In order to cover a broad range of topics and magazines, the only search filter that we used was chosen to ensure that the subjects were related to data science and/or artificial intelligence.

**Figure 1:**
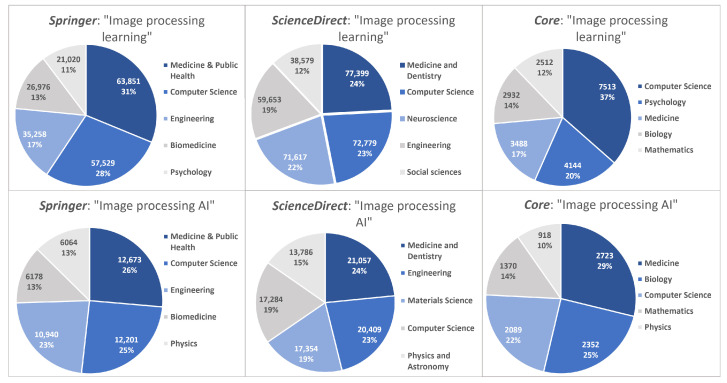
Main research areas for the tested search inputs for three different academic engines.

As of February 2023, a search using the prompt “image processing AI” returns manuscripts related mostly to “Medicine”, “Computer science”, and “Engineering”. In fact, while searching in the three different research aggregators, the results stayed somewhat consistent. A summary of the results obtained can be observed in Figure 1.

Since there is more research available on some topics, the cases described ahead can also have a higher prevalence when compared to others.

### 2.2. Inclusion and Exclusion Criteria for Article Selection

The research carried out in the different repositories resulted in a large number of research works proposed by different authors. By considering the constant advances made in this subject and the amount of research developed, we opted to mainly focus on research developed in the last 5 years. We analyzed and selected the research sources that provided novel and/or interesting applications of ML in image processing. The objective was to present a broad representation of the recent trends in ML research and provide the information in a more concise form.

## 3. Technical Background

The growing use of the internet in general, and social networks in particular, has led to the availability of a large increase in digital images; being privileged means being able to express emotions and share information, which enables many diverse applications [10]. Identifying the interesting parts of a scene is a fundamental step for recognizing and interpreting an image [11]. To understand how the different techniques are applied in processing images and extracting their features, as well as explaining the main concepts and technicalities of the different types of AI models, we will provide a general technical background review of machine learning and image processing, which will help provide relevant context to the scope of this review, as well as guide the reader through the covered topics.

### 3.1. Graphics Processing Units

Many advances covered in this paper, along with classical ML and scalable general-purpose graphics processing unit (GPU) computing, have become critical components of AI [1,10], enabling the processing of massive amounts of data generated each day and lowering the barrier to adoption [1]. In particular, the usage of GPUs revolutionized the landscape of classical ML and DL models. From the 1990s to the late 2000s, ML research was predominantly focused on SVM, which was considered state-of-the-art [1]. In the following decade, starting in 2010, GPUs brought new life into the field of DL, jumpstarting a high amount of research and development [1]. State-of-the-art DL algorithms tend to have higher computational complexity, requiring several iterations to make the parameters converge to an optimal value [12,13]. However, the relevance of DL has only become greater over the years, as this technology has gradually become one of the main focuses of ML research [14].

While research into the use of ML on GPUs predates the recent resurgence of DL, the usage of general-purpose GPUs for computing (GPGPU) became widespread when CUDA was released in 2007 [1]. Shortly after, CNN started to be implemented on top of GPUs, demonstrating dramatic end-to-end speedup, even over highly optimized CPU implementations. CNNs are a subset of methods that can be used, for example, for image restoration, which has demonstrated outstanding performance [1]. Some studies have shown that, when compared with traditional neural networks and SVM, the accuracy of recognition using CNNs is notably higher [12].

Some of these performance gains were accomplished even before the existence of dedicated GPU-accelerated BLAS libraries. The release of the first CUDA Toolkit brought new life to general-purpose parallel computing with GPUs, with one of the main benefits of this approach being the ability of GPUs to enable a multithreaded single-instruction (SIMT) programming paradigm, higher throughput, and more parallel models when compared to SIMD. This process makes several blocks of multiprocessors available, each with many parallel cores (threads), allowing access to high-speed memory [1].

### 3.2. Image Processing

For humans, an image is a visual and meaningful arrangement of regions and objects [11]. Recent advances in image processing methods find application in different contexts of our daily lives, both as citizens and in the professional field, such as compression, enhancement, and noise removal from images [10,15]. In classification tasks, an image can be transformed into millions of pixels, which makes data processing very difficult [2]. As a complex and difficult image-processing task, segmentation has high importance and application in several areas, namely in automatic visual systems, where precision affects not only the segmentation results but also the results of the following tasks, which, directly or indirectly, depend on it [11]. In segmentation, the goal is to divide an image into its constituent parts (or objects)—sometimes referred to as regions of interest (ROI)—without overlapping [16,17], which can be achieved through different feature descriptors, such as the texture, color, and edges, as well as a histogram of oriented gradients (HOG) and a global image descriptor (GIST) [11,17]. While the human vision system segments images on a natural basis, without special effort, automatic segmentation is one of the most complex tasks in image processing and computer vision [16].

Given its high applicability and importance, object detection has been a subject of high interest in the scientific community. Depending on the objective, it may be necessary to detect objects with a significant size compared to the image where they are located or to detect several objects of different sizes. The results of object detection in images vary depending on their dimensions and are generally better for large objects [18]. Image processing techniques and algorithms find application in the most diverse areas. In the medical field, image processing has grown in many directions, including computer vision, pattern recognition, image mining, and ML [19].

In order to use some ML models when problems in image processing occur, it is often necessary to reduce the number of data entries to quickly extract valuable information from the data [10]. In order to facilitate this process, the image can be transformed into a reduced set of features in an operation that selects and measures the representative data properties in a reduced form, representing the original data up to a certain degree of precision, and mimicking the high-level features of the source [2]. While deep neural networks (DNNs) are often used for processing images, some traditional ML techniques can be applied to improve the data obtained. For example, in Zeng et al. [20], a deep convolutional neural network (CNN) was used to extract image features, and principal component analysis (PCA) was applied to reduce the dimensionality of the data.

### 3.3. Machine Learning Overview

ML draws inspiration from a conceptual understanding of how the human brain works, focusing on performing specific tasks that often involve pattern recognition, including image processing [1], targeted marketing, guiding business decisions, or finding anomalies in business processes [4]. Its flexibility has allowed it to be used in many fields owing to its high precision, flexible customization, and excellent adaptability, being increasingly more common in the fields of environmental science and engineering, especially in recent years [3]. When learning from data, deep learning systems acquire the ability to identify and classify patterns, making decisions with minimal human intervention [2]. Classical techniques are still fairly widespread across different research fields and industries, particularly when working with datasets not appropriate for modern deep learning (DL) methods and architectures [1]. In fact, some data scientists like to reinforce that no single ML algorithm fits all data, with proper model selection being dependent on the problem being solved [21,22]. In diagnosis modeling that uses the classification paradigm, the learning process is based on observing data as examples. In these situations, the model is constructed by learning from data along with its annotated labels [2].

While ML models are an important part of data handling, other steps need to be taken in preparation, like data acquisition, the selection of the appropriate algorithm, model training, and model validation [3]. The selection of relevant features is one of the key prerequisites to designing an efficient classifier, which allows for robust and focused learning models [23].

There are two main classes of methods in ML: supervised and unsupervised learning, with the primary difference being the presence of labels in the datasets.

- In supervised learning, we can determine predictive functions using labeled training datasets, meaning each data object instance must include an input for both the values and the expected labels or output values [21]. This class of algorithms tries to identify the relationships between input and output values and generate a predictive model able to determine the result based only on the corresponding input data [3,21]. Supervised learning methods are suitable for regression and data classification, being primarily used for a variety of algorithms like linear regression, artificial neural networks (ANNs), decision trees (DTs), support vector machines (SVMs), k-nearest neighbors (KNNs), random forest (RF), and others [3]. As an example, systems using RF and DT algorithms have developed a huge impact on areas such as computational biology and disease prediction, while SVM has also been used to study drug–target interactions and to predict several life-threatening diseases, such as cancer or diabetes [23].
- Unsupervised learning is typically used to solve several problems in pattern recognition based on unlabeled training datasets. Unsupervised learning algorithms are able to classify the training data into different categories according to their different characteristics [21,24], mainly based on clustering algorithms [24]. The number of categories is unknown, and the meaning of each category is unclear; therefore, unsupervised learning is usually used for classification problems and for association mining. Some commonly employed algorithms include K-means [3], SVM, or DT classifiers. Data processing tools like PCA, which is used for dimensionality reduction, are often necessary prerequisites before attempting to cluster a set of data.

Some studies make reference to semi-supervised learning, in which a combination of unsupervised and supervised learning methods are used. In theory, a mixture of labeled and unlabeled data is used to help reduce the costs of labeling a large amount of data. The advantage is that the existence of some labeled data should make these models perform better than strictly unsupervised learning [21].

In addition to the previously mentioned classes of methods, reinforcement learning (RL) can also be regarded as another class of machine learning (ML) algorithms. This class refers to the generalization ability of a machine to correctly answer unlearned problems [3].

The current availability of large amounts of data has revolutionized data processing and statistical modeling techniques but, in turn, has brought new theoretical and computational challenges. Some problems have complex solutions due to scale, high dimensions, or other factors, which might require the application of multiple ML models [4] and large datasets [25]. ML has also drawn attention as a tool in resource management to dynamically manage resource scaling. It can provide data-driven methods for future insights and has been regarded as a promising approach for predicting workload quickly and accurately [26]. As an example, ML applications in biological fields are growing rapidly in several areas, such as genome annotation, protein binding, and recognizing the key factors of cancer disease prediction [23]. The deployment of ML algorithms on cloud servers has also offered opportunities for more efficient resource management [26].

Most classical ML techniques were developed to target structured data, meaning data in a tabular form with data objects stored as rows and the features stored as columns. In contrast, DL is specifically useful when working with larger, unstructured datasets, such as text and images [1]. Additional hindrances may apply in certain situations, as, for example, in some engineering design applications, heterogeneous data sources can lead to sparsity in the training data [25]. Since modern problems often require libraries that can scale for larger data sizes, a handful of ML algorithms can be parallelized through multiprocessing. Nevertheless, the final scale of these algorithms is still limited by the amount of memory and number of processing cores available on a single machine [1].

Some of the limitations in using ML algorithms come from the size and quality of the data. Real datasets are a challenge for ML algorithms since the user may face skewed label distributions [1]. Such class imbalances can lead to strong predictive biases, as models can optimize the training objective by learning to predict the majority label most of the time. The term “ensemble techniques” in ML is used for combinations of multiple ML algorithms or models. These are known and widely used for providing stability, increasing model performance, and controlling the bias-variance trade-off [1]. Hyperparameter tuning is also a fundamental use case in ML, which requires the training and testing of a model over many different configurations to be able to find the model with the best predictive performance. The ability to train multiple smaller models in parallel, especially in a distributed environment, becomes important when multiple models are being combined [1].

Over the past few years, frequent advances have occurred in AI research caused by a resurgence in neural network methods that have fueled breakthroughs in areas like image understanding, natural language processing, and others [27]. One area of AI research that appears particularly inviting from this perspective is deep reinforcement learning (DRL), which marries neural network modeling with RL techniques. This technique has exploded within the last 5 years into one of the most intense areas of AI research, generating very promising results to mimic human-level performance in tasks varying from playing poker [28], video games [29], multiplayer contests, and complex board games, including Go and Chess [27]. Beyond its inherent interest as an AI topic, DRL might hold special interest for research in psychology and neuroscience since the mechanisms that drive learning in DRL were partly inspired by animal conditioning research and are believed to relate closely to neural mechanisms for reward-based learning centering on dopamine [27].

#### 3.3.1. Deep Learning Concepts

**Figure 2:**
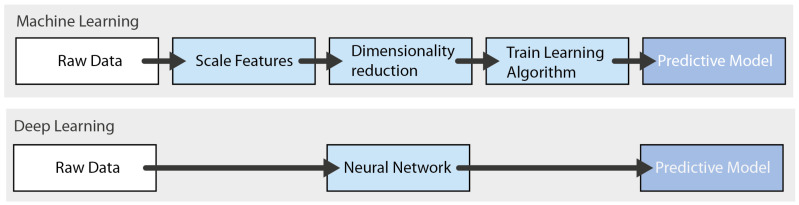
Differences in the progress stages between traditional ML methods and DL methods.

DL is a heuristic learning framework and a sub-area of ML that involves learning patterns in data structures using neural networks with many nodes of artificial neurons called perceptrons [10,19,30] (see Figure 2). Artificial neurons can take several inputs and work according to a mathematical calculation, returning a result in a process similar to a biological neuron [19]. The simplest neural network, known as a single-layer perceptron [30], is composed of at least one input, one output, and a processor [31]. Three different types of DL algorithms can be differentiated: multilayered perceptron (MLP) with more than one hidden layer, CNN, and recurrent neural networks (RNNs) [32].

One important consideration towards generic neural networks is they are extremely low-bias learning systems. As dictated by the bias–variance trade-off, this means that neural networks, in the most generic form employed in the first DRL models, tend to be sample-inefficient and require large amounts of data to learn. A narrow hypothesis set can speed the learning process if it contains the correct hypothesis or if the specific biases the learner adopts happen to fit with the material to be learned [27]. Several proposals for algorithms and models have emerged, some of which have been extensively used in different contexts, such as CNNs, autoencoders, and multilayer feedback RNN [10]. For datasets of images, speech, and text, among others, it is necessary to use different network models in order to maximize system performance [33]. DL models are often used for image feature extraction and recognition, given their higher performance when dealing with some of the traditional ML problems [10].

DL techniques differ from traditional ML in some notable ways (see also Figure 2):

1. Training a DNN implies the definition of a loss function, which is responsible for calculating the error made in the process given by the difference between the expected output value and that produced by the network. One of the most used loss functions in regression problems is the mean squared error (MSE) [30]. In the training phase, the weight vector that minimizes the loss function is adjusted, meaning it is not possible to obtain analytical solutions effectively. The loss function minimization method usually used is gradient descent [30].
2. Activation functions are fundamental in the process of learning neural network models, as well as in the interpretation of complex nonlinear functions. The activation function adds nonlinear features to the model, allowing it to represent more than one linear function, which would not happen otherwise, no matter how many layers it had. The Sigmoid function is the most commonly used activation function in the early stages of studying neural networks [30].
3. As their capacity to learn and adjust to data is greater than that of traditional ML models, it is more likely that overfitting situations will occur in DL models. For this reason, regularization represents a crucial and highly effective set of techniques used to reduce the generalization errors in ML. Some other techniques that can contribute to achieving this goal are increasing the size of the training dataset, stopping at an early point in the training phase, or randomly discarding a portion of the output of neurons during the training phase [30].
4. In order to increase stability and reduce convergence times in DL algorithms, optimizers are used, with which greater efficiency in the hyperparameter adjustment process is also possible [30].

In the last decades, three main mathematical tools have been studied for image modeling and representation, mainly because of their proven modeling flexibility and adaptability. These methods are the ones based on probability statistics, wavelet analysis, and partial differential equations [34,35]. In image processing procedures, it is sometimes necessary to reduce the number of input data. An image can be translated into millions of pixels for tasks, such as classifications, meaning that data entry would make the processing very difficult. In order to overcome some difficulties, the image can be transformed into a reduced set of features, selecting and measuring some representative properties of raw input data in a more reduced form [2]. Since DL technologies can automatically mine and analyze the data characteristics of labeled data [13,14], this makes DL very suitable for image processing and segmentation applications [14]. Several approaches use autoencoders, a set of unsupervised algorithms, for feature selection and data dimensionality reduction [31].

**Figure 3:**
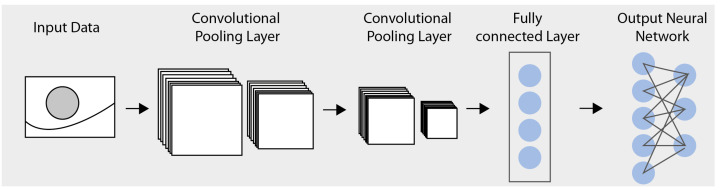
Illustration of the structure of a CNN.

Among the many DL models, CNNs have been widely used in image processing problems, proving more powerful capabilities in image processing than traditional algorithms [36]. As shown in Figure 3, a CNN, like a typical neural network, comprises an input layer, an output layer, and several hidden layers [37]. A single hidden layer in a CNN typically consists of a convolutional layer, a pooling layer, a fully connected layer [38], and a normalization layer.

Additionally, the number of image-processing applications based on CNNs is also increasing daily [10]. Among the different DL structures, CNNs have proven to be more efficient in image recognition problems [20]. On the other hand, they can be used to improve image resolution, enhancing their applicability in real problems, such as the transmission or storage of images or videos [39].

DL models are frequently used in image segmentation and classification problems, as well as object recognition and image segmentation, and they have shown good results in natural language processing problems. As an example, face recognition applications have been extensively used in multiple real-life examples, such as airports and bank security and surveillance systems, as well as mobile phone functionalities [10].

There are several possible applications for image-processing techniques. There has been a fast development in terms of surveillance tools like CCTV cameras, making inspecting and analyzing footage more difficult for a human operator. Several studies show that human operators can miss a significant portion of the screen action after 20 to 40 minutes of intensive monitoring [18]. In fact, object detection has become a demanding study field in the last decade. The proliferation of high-powered computers and the availability of high-speed internet has allowed for new computer vision-based detection, which has been frequently used, for example, in human activity recognition [18], marine surveillance [40], pedestrian identification [18], and weapon detection [41].

One alternative application of ML in image-processing problems is image super-resolution (SR), a family of technologies that involve recovering a super-resolved image from a single image or a sequence of images of the same scene. ML applications have become the most mainstream topic in the single-image SR field, being effective at generating a high-resolution image from a single low-resolution input. The quality of training data and the computational demand remain the two major obstacles in this process [42].

#### 3.3.2. Reinforcement Learning Concepts

RL is a set of ML algorithms that use a mathematical framework that can learn to optimize control strategies directly from the data [4,43] based on a reward function in a Markov decision process [44,45]. The Markov decision process (MDP) is a stochastic process used to model the decision-making process of a dynamic system. The decision process is sequential, where actions/decisions depend on the current state and the system environment, influencing not only the immediate rewards but also the entire decision process [4]. One commonly referenced RL problem is *the multi-armed bandit*, in which an agent selects one of *n* different options and receives a reward depending on the selection. This problem illustrates how RL can provide a trade-off between exploration (trying different arms) and exploitation (playing the arm with the best results) [44]. This group of algorithms is derived from behaviorist psychology, where an intelligent body explores the external environment and updates its strategy with feedback signals to maximize the cumulative reward [43], which means the action is exploitative [46].

In RL, the behavior of the Markov decision process is determined by a reward function [4]. The basis of a DRL network is made up of an agent and an environment, following an action-reward type of operation. The interaction begins in the environment with the sending of its state to the agent, which takes an action consistent with the state received, according to which it is subsequently rewarded or penalized by the environment [4,44,46,47,48]. RL is considered an autonomous learning technique that does not require labeled data but for which search and value function approximation are vital tools [4]. Often, the success of RL algorithms depends on a well-designed reward function [45]. Current RL methods still present some challenges, namely the efficiency of the learning data and the ability to generalize to new scenarios [49]. Nevertheless, this group of techniques has been used with tremendous theoretical and practical achievements in diverse research topics such as robotics, gaming, biological systems, autonomous driving, computer vision, healthcare, and others [44,48,50,51,52,53].

One common technique in RL is random exploration, where the agent makes a decision on what to do randomly, regardless of its progress [46]. This has become impractical in some real-world applications since learning times can often become very large. Recently, RL has shown a significant performance improvement compared to non-exploratory algorithms [46,54]. Another technique, inverse reinforcement learning (IRL), uses an opposite strategy by aiming to find a reward function that can explain the desired behavior [45]. In a recent study using IRL, Hwang et al. [45] proposed a new RL method, named *option compatible reward inverse reinforcement learning*, which applies an alternative framework to the compatible reward method. The purpose was to assign reward functions to a hierarchical IRL problem that is introduced while making the knowledge transfer easier by converting the information contained in the options into a numerical reward value. While the authors concluded that their novel algorithm was valid in several classical benchmark domains, they remarked that applying it to real-world problems still required extended evaluation.

RL models have been used in a wide variety of practical applications. For example, the COVID-19 pandemic was one of the health emergencies with the widest impact that humans have encountered in the past century. Many studies were directed towards this topic, including many that used ML techniques to several effects. Zong and Luo (2022) [55] conducted a study where they employed a custom epidemic simulation environment for COVID-19 where they applied a new multi-agent RL-based framework to explore optimal lockdown resource allocation strategies. The authors used real epidemic transmission data to calibrate the employed environment to obtain results more consistent with the real situation. Their results indicate that the proposed approach can adopt a flexible allocation strategy according to the age distribution of the population and economic conditions. These insights could be extremely valuable for decision-makers in supply chain management.

Some technical challenges blocked the combination of DNN with RL until 2015, when breakthrough research demonstrated how the integration could work in complex domains, such as Atari video games [29,56], leading to rapid progress toward improving and scaling DRL [27]. Some of the first successful applications of DRL came with the success of the deep Q network algorithm [56]. Currently, the application of DRL models to computer vision problems, such as object detection and tracking or image segmentation, has gained emphasis, given the good results it has produced [31]. RL, along with supervised and unsupervised methods, are the three main pattern recognition models used for research [57].

The initial advances in RL were boosted by the good performance of the [56] replay algorithm, as well as the use of two networks, one with fixed weights, which serves as the basis for a second network, for which the weights are iteratively updated during training, replacing the first one when the learning process ends. With the aim of reducing the high convergence times of DRL algorithms, several distributed framework approaches [58] have been proposed. This suit of methods has been successfully used for applications in computer vision [59] and in robotics [58].

### 3.4. Current Challenges

Considering everything that has been discussed previously, some of the main challenges that AI image processing faces are common across multiple subjects. Most applications require a large volume of images that are difficult to obtain. Indeed, due to the large amount of data, the process of extracting features from a dataset can become very time and resource-consuming. Some models, such as CNNs, can potentially have millions of parameters to be learned, which might require considerable effort to obtain sufficient labeled data [60]. Since AI models are heavily curated for a given purpose, the model obtained will likely be inapplicable outside of the specific domain in which it was trained. The performance of a model can be heavily impacted by the data available, meaning the accuracy of the outcome can also vary heavily [61]. An additional limitation that has been identified during research is the sensitivity of models regarding noisy or biased data [60]. A meticulous and properly designed data-collection plan is essential, often complemented by a prepossessing phase to ensure good-quality data. Some researchers have turned their attention to improving the understanding of the many models. Increased focus has been placed on the way the weights of a neural network can sometimes be difficult to decipher and extract useful information from, which can lead to wrong assumptions and decisions [62]. In order to facilitate communication and discussion, some authors have also attempted to provide a categorization system of DL methodologies based on their applications [31].

## 4. Image Processing Developments

**Figure 4:**
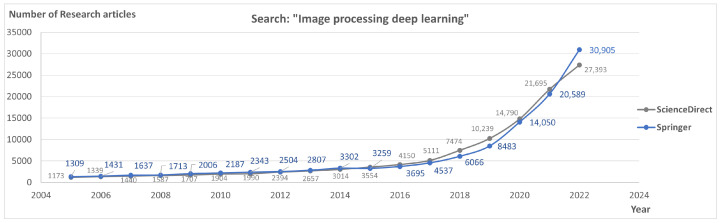
Number of research articles found using the search query “image processing deep learning” for two different aggregators.

The topic of ML has been studied with very broad applications and in multiple areas that require data collection and processing. Considering recent publications from the last 7 years (2017–2023), we see that several studies have been developed dealing with different subjects, with proposals of many different models. In particular, we found a considerable amount of research papers showing interest in using DL in medicine, engineering, and biology. When we consider the volume of research developed, there is a clear increase in published research papers targeting image processing and DL, over the last decades. A search using the terms “image processing deep learning” in *Springerlink* generated results demonstrating an increase from 1309 articles in 2005 to 30,905 articles in 2022, only considering review and research papers. In the aggregator *Science Direct*, we saw a similar result, demonstrating an increase from 1173 in 2005 to 27,393 scientific manuscripts in 2022. The full results across the referred timeline can be observed in Figure 4. These results validate an upward trend in attention to DL methods, as also described in the previous section.

A lot of recent literature, especially in the medical field, has attempted to address the biggest challenges, mainly derived from data scarcity and model performance [14,61,62,63,64]. Some research has focused on improving perforce or reducing the computational requirements in models such as CNNs [60,65,66] using techniques such as model pruning or compression. These have the objective of reducing the model’s overall size or operating cost. In the next section, we will discuss relevant approaches taken on the subject to illustrate how the scientific community has been using ML methods to solve specific data-driven problems and discuss some of the implications.

### 4.1. Domains

Studies involving image processing can be found on topics such as several infrastructure monitoring applications [13,67,68] in road pavement [69,70,71], remote sensing images [12], image reconstruction [72], detecting and quantifying plant diseases [73,74,75,76,77], identification of pests in plant crops [17,78,79], automated bank cheque verification [80] or even for graphical search [11,81,82,83]. There is also an ample amount of research using ML algorithms in the medical field. DL techniques have been applied in infection monitoring [64,84,85], in developing personalized advice for treatment [19,86], in diagnosing several diseases like COVID-19 [63,87,88,89], or imaging procedures including radiology [14,63,90,91] and pathology imaging [19] or in cancer screening [91,92,93,94].

While most modern research hasn’t focused on traditional ML techniques, there are still some valuable lessons to be taken from these studies, with interesting results obtained in engineering subjects. In 2022, Pratap and Sardana [21] conducted and published a review on image processing in materials science and engineering using ML. In this study, the authors reviewed several research materials focusing on ML, the ML model selection, and the image processing technique used, along with the context of the problem. The authors suggested *SimpleCV* as a possible framework, specifically for digital image processing. This type of approach was justified by the authors since materials have a 3D structure but most of the analysis on image processing that has been done is of 2D images [21]. Image super-resolution (SR) is another interesting application of ML concepts for image processing challenges that has attracted some attention in the past decades [15,42]. In 2016, Zhao et al. [42] proposed a framework for single-image super-resolution tasks, consisting of kernel blur estimation, to improve the training quality as well as the model performance. Using the kernel blur estimation, the authors adopted a selective patch processing strategy combined with sparse recovery. While their result indicated a better level of performance than several super-resolution approaches, some of the optimization problems encountered were, themselves, extraordinarily time-consuming, and as such, not a suitable solution for efficiency improvement. Research such as those can often serve as inspiration to address nuanced engineering problems that may be more specific to certain research subjects. As an example, in the last decade, the automobile industry has made a concerted shift towards intelligent vehicles equipped with driving assistance systems, with new vision systems in some higher-end cars. Some vision systems include cameras mounted in the car, which can be used by engineers to obtain large quantities of images and develop many of the future self-driving car functionalities [66].

Some advanced driver assistance systems (ADAS) that use AI have been proposed to assist drivers and attempt to significantly decrease the number of accidents. These systems often employ technologies such as image sensors, global positioning, radar imaging, and computer vision techniques. Studies have been developed that tested a number of different image processing techniques to understand their accuracy and limitations and found good results with traditional ML methods like SVM and optimum-path forest classifier [95] or K-Means clustering [11]. One potential benefit of using this approach is that some traditional methods can be less costly to apply and can be used as complementary on many different subjects. Rodellar et al. [16] investigated the existing research on the analysis of blood cells, using image processing. The authors acknowledged the existence of subtle morphological differences for some lymphoma and leukemia cell types, that are difficult to identify in routine screening. Some of their most curious findings were that the methods most commonly used in the classification of PB cells were Neural Networks, Decision Trees (DT), and SVM. The authors noted that image-based automatic recognition systems could position themselves as new modules of the existing analyzers or even that new systems could be built and combined with other well-established ones.

#### 4.1.1. Research Using Deep Learning

Regarding Deep Learning methodologies, many studies attempt to improve the performance of DL models, which we highlight next. In their research, Monga et al. [96] conducted a review of usage and research involving Deep Neural Networks (DNN) that covered some of the most popular techniques for algorithm unrolling in several domains of signal and image processing. The authors extensively covered research developed on a technique called algorithm unrolling or unfolding. This method can provide a concrete and systematic connection between iterative algorithms, which are used widely in signal processing, and DNNs. This type of application has recently attracted enormous attention both in theoretical investigations and practical applications. The authors noted that while substantial progress has been made, more work needs to be done to comprehend the mechanism behind the unrolling network behavior. In particular, they highlight the need to clarify why some of the state-of-the-art networks perform so well on several recognition tasks. In a study published by Zeng et al. [20], a correction neural network model named *Boundary Regulated Network* (BR-Net) was proposed. It used high-resolution remote satellite images as the source, and the features of the image were extracted through convolution, pooling, and classification. The model accuracy was additionally increased through training on the experimental dataset in a particular area. In their findings, the authors indicated a performance improvement of 15%, while the recognition speed was also increased by 20%, compared with the newly researched models, further noting that, for a considerably large amount of data, the model will have poor generalization ability. In Farag [66], the investigation focused on the ability of a CNN model to learn safe driving maneuvers based on data collected using a front-facing camera. Their data collection happened using urban routes and was performed by an experienced driver. The author developed a 17-layer behavior cloning CNN model with four drop-out layers added to prevent overfitting during training. The results looked promising enough, whereby a small amount of training data from a few tracks was sufficient to train the car to drive safely on multiple tracks. For such an approach, one possible shortcoming is that the approach taken may require a massive number of tracks in order to be able to generalize correctly for actual street deployment.

Some modern research has focused on expanding the practical applications of DL models in image processing:

- One of the first DL models used for video prediction, inspired by the sequence-to-sequence model usually used in natural language processing [97], uses a recurrent long and short term memory network (LSTM) to predict future images based on a sequence of images encoded during video data processing [97].
- In their research, Salahzadeh et al. [98] presented a novel mechatronics platform for static and real-time posture analysis, combining 3 complex components. The components included a mechanical structure with cameras, a software module for data collection and semi-automatic image analysis, and a network to provide the raw data to the DL server. The authors concluded that their device, in addition to being inexpensive and easy to use, is a method that allows postural assessment with great stability and in a non-invasive way, proving to be a useful tool in the rehabilitation of patients.
- Studies in graphical search engines and content-based image retrieval (CBIR) systems have also been successfully developed recently [11,82,99,100], with processing times that might be compatible with real-time applications. Most importantly, the corresponding results of these studies appeared to show adequate image retrieval capabilities, displaying an undisputed similarity between input and output, both on a semantic basis and a graphical basis [82]. In a review by Latif et al. [101], the authors concluded that image feature representation, as it is performed, is impossible to be represented by using a unique feature representation. Instead, it should be achieved by a combination of said low-level features, considering they represent the image in the form of patches and, as such, the performance is increased.
- In their publication, Rani et al. [102] reviewed the current literature found on this topic from the period from 1995 to 2021. The authors found that researchers in microbiology have employed ML techniques for the image recognition of four types of micro-organisms: bacteria, algae, protozoa, and fungi. In their research work, Kasinathan and Uyyala [17] apply computer vision and knowledge-based approaches to improve insect detection and classification in dense image scenarios. In this work, image processing techniques were applied to extract features, and classification models were built using ML algorithms. The proposed approach used different feature descriptors, such as texture, color, shape, histograms of oriented gradients (HOG) and global image descriptors (GIST). ML was used to analyze multivariety insect data to obtain the efficient utilization of resources and improved classification accuracy for field crop insects with a similar appearance.

As the most popular research area for image processing, research studies using DL in the medical field exist in a wide variety of subjects. Automatic classifiers for imaging purposes can be used in many different medical subjects, often with very good results. However, the variety of devices, locations, and sampling techniques used can often lead to undesired or misunderstood results. One clear advantage of these approaches is that some exams and analyses are based on a human inspection, which can be time-consuming, require extensive training for the personnel, and may also be subject to subjectivity and variability in the observers [16,103,104]. In 2023, Luis et al. applied *explainable artificial intelligence (xAI)* as a way to test the application of different classifiers for monkeypox detection and to better understand the results [62]. With a greater focus on properly interpreting the model results, approaches such as these are increasingly more common. Recently, Melanthota et al. [32] conducted a review of research regarding DL-based image processing in optical microscopy. DL techniques can be particularly useful in this topic since manual image analysis of tissue samples tends to be a very tedious and time-consuming process due to the complex nature of the biological entities, while the results can also be highly subjective. The authors concluded that DL models perform well in improving image resolution in smartphone-based microscopy, being an asset in the development and evolution of healthcare solutions in remote locations. The authors also identified an interesting application of DL to monitor gene expression and protein localization in organisms. Overall, it was noted how CNN-based DL networks have emerged as a model with great potential for medical image processing.

Brain image segmentation is a subject addressed by a vast number of researchers who seek to develop systems for accurate cancer diagnosis able to differentiate cancer cells from healthy ones [105,106,107,108,109,110,111]. A problem that such approaches can mitigate is that human verification of magnetic resonance imaging to locate tumors can be prone to errors. In a recent study, Devunooru et al. [105] provided a taxonomy system for the key components needed to develop an innovative brain tumor diagnosis system based on DL models. The taxonomy system, named *data image segmentation processing and viewing* (DIV), comprised research that had been developed since 2016. The results indicated that the majority of the proposed approaches only applied two factors from the taxonomy system, namely data and image segmentation, ignoring a third important factor, which is "view". The comprehensive framework developed by the authors considers all three factors to overcome the limitations of state-of-the-art solutions. Finally, the authors consider that efforts should be made to increase the efficiency of approaches used in image segmentation problems, as well as in problems processing large quantities of medical images.

In their review, Yedder et al. [112] focused on studying state-of-the-art medical image reconstruction algorithms focused on DL-based methods. The main focus of his research was the reconstruction of biomedical images as an important source of information for the elaboration of medical diagnoses. The authors’ work focused on the differences observed by applying conventional reconstruction methods in contrast to learning-based methods. They showed particular interest in the success of DL in computer vision and medical imaging problems, as well as its recent rise in popularity, concluding that DL-based methods appeared to adequately address the noise sensitivity and the computational inefficiency of iterative methods. Furthermore, the authors noted that the use of DL methods in medical image reconstruction encompassed an ever-increasing number of modalities, noting a clear trend in the newer art toward unsupervised approaches, primarily instigated by the constraints in realistic or real-world training data.

#### 4.1.2. Research Using Reinforcement Learning

Finally, we will finalize our state-of-the-art review by referencing research that used reinforcement learning approaches, mostly in combination with deep learning methods. RL research has been developed in several topics, including robotics [113,114,115], design automation [25], energy management strategies for hybrid vehicles [43], parameter estimation in the context of biological systems [44,116,117], in facial motion learning [48,50,118], and have also been successfully applied in closed-world environments, such as games [51,54,119,120]. In the topic of image processing, some pertinent studies were found, especially using DRL [31,47,57,121]. Many novel applications continue to be proposed by researchers. A study conducted in 2022 by Dai et al. [122] explored effective healthcare strategies for simulated human bodies through the combination of DRL methods with conceptual embedding techniques. In this instance, the DNN architecture was used to recreate the transformation function of the input-output characteristics in a human body, using a dataset containing 990 tongue images of nine body constitution (BC) types. The authors concluded that the proposed framework could be particularly useful when applied to a high-dimensional dynamic system of the human body. Amongst the most relevant research encountered, we highlight the following:

In order to overcome the challenges in computer vision, in terms of data-efficiency or generalizing to new environments, a study from 2020 by Laskin et al. [49] presented a reinforcement learning module with augmented data leveraging, which could be incorporated in typical RL systems to effortlessly improve their overall performance. The authors remarked that data augmentations could potentially improve data efficiency in RL methods operating from pixels, even without significant changes to the underlying RL algorithm. The proposed approach by Laskin et al. [49] could help make deep RL be more practical for solving real-world problems. In a different example, Khayyat and Elrefaei Khayyat and Elrefaei [47] successfully developed a system for retrieving ancient images from Arabic manuscripts through an RL agent. The main benefit of this approach was the reduction of data dimensionality, which leads to increased accuracy in image classification and retrieval tasks. Image visual features, extracted using a pre-trained VGG19 convolutional neural network, are fused with textual features through a concatenation and hash merge layer. The success achieved in this scenario may also suggest that the model can be applied to other types of images.

Amongst the recent advancements in DRL focusing on computing optimization is the work presented by Ren et al. [57], which proposed a system for image stereo-matching algorithms with rule constraints and parallax estimation. Initially, the edge pixel constraint rules were established, and adjustments were made to the image blocks; then, the image parallax estimation was performed, and a DRL analysis was executed by a CNN in an iterative way. The results showed the proposed algorithm was able to complete convergence quickly, with an accuracy of up to more than 95%. However, the matching targets were not clearly defined, particularly in small objects with curved surfaces, which could limit their practicality. Due to a large number of existing models, in 2022, Le et al. [31] conducted an extensive review of the state-of-the-art advances using DRL in computer vision research. The main objective was to propose a categorization of DRL methodologies, present the potential advantages and limitations in computer vision, and discuss future research directions. The authors propose to divide DRL methods into seven categories, depending on their applications: (i) landmark localization, (ii) object detection, (iii) object tracking, (iv) registration on both 2D image and 3D image volumetric data, (v) image segmentation, (vi) video analysis, and (vii) other applications. Some of the most promising approaches selected by the authors to create new insights into this research field included inverse DRL, multi-agent DRL, meta DRL, and imitation learning.

## 5. Discussion and Future Directions

Although the advances and successes of ML are undeniable, particularly in the field of digital image processing, there are still important limitations, both in terms of its operational mode and in terms of its design. One of the most important is the fact that, for the most part, the algorithms developed to date are trained to perform a certain task, being able to solve a particular problem. The generalization capacity of existing ML models is limited, making it difficult to apply them to solve problems other than those for which they were trained. Although it is possible to apply learning transfer techniques with the aim of using existing models in new contexts, the results still fall short of the needs.

As previously noted, another one of the limitations we identified concerns the models’ efficiency. ML, in particular DL techniques, requires a large amount of data and computational resources to train and run the models, which may be infeasible or impractical in some scenarios or applications. This requires techniques that can reduce the cost and time of training and inference, as well as increase the robustness and generalization of the models. Some examples of these techniques are model compression, model pruning, model quantization, and knowledge distillation, among others.

Additionally, it is important to highlight the difficulty in interpreting DL models, given their complexity and opacity, which makes it difficult to understand their internal functioning, as well as the results produced. This requires techniques that can explain the functioning, logic, and reliability of models, as well as the factors that influence their decisions. Some examples of these techniques are the visualization of activations, sensitivity analysis, attribution of importance, and generation of counterfacts, among others.

No less important are the limitations that deserve some reflection related to ethics and responsibility since DL has a major impact on society, business, and people. This requires the use of techniques that can guarantee the privacy, security, transparency, justice, and accountability of models, as well as avoid or mitigate their possible negative effects. Some examples of techniques that can help in the mitigation of such limitations are homomorphic encryption, federated learning, algorithmic auditing, and bias detection.

## 6. Conclusions

In this review, we analyzed some of the most recent works developed in ML, particularly using DL and RL methods or combinations of these. It is becoming increasingly obvious that image processing systems are applied in the most diverse contexts and have seen increasingly more impressive results as the methods have matured. Some of the observed trends appear to indicate a prevalence of certain techniques in certain research topics, which is not surprising. Amongst these trends, we observed:

- Interest in image-processing systems using DL methods has exponentially increased over the last few years. The most common research disciplines for image processing and AI are medicine, computer science, and engineering.
- Traditional ML methods are still extremely relevant and are frequently used in fields such as computational biology and disease diagnosis and prediction or to assist in specific tasks when coupled with other more complex methods. DL methods have become of particular interest in many image-processing problems, particularly because of their ability to circumvent some of the challenges that more traditional approaches face.
- A lot of attention from researchers seems to focus on improving model performance, reducing computational resources and time, and expanding the application of ML models to solve concrete real-world problems.
- The medical field seems to have developed a particular interest in research using multiple classes and methods of learning algorithms. DL image processing has been useful in analyzing medical exams and other imaging applications. Some areas have also still found success using more traditional ML methods.
- Another area of interest appears to be autonomous driving and driver profiling, possibly powered by the increased access to information available both for the drivers and the vehicles alike. Indeed, modern driving assistance systems have already implemented features such as (a) road lane finding, (b) free driving space finding, (c) traffic sign detection and recognition, (d) traffic light detection and recognition, and (e) road-object detection and tracking. This research field will undoubtedly be responsible for many more studies in the near future.
- Graphical search engines and content-based image retrieval systems also present themselves as an interesting topic of research for image processing, with a diverse body of work and innovative approaches.

We found interesting applications using a mix of DL and RL models. The main advantage of these approaches is having the potential of DL to process and classify the data and use reinforcement methods to capitalize on the historical feedback of the performed actions to fine-tune the learning hyperparameters. This is one area that seems to have become a focus point of research, with an increasing number of studies being developed in an area that is still recent. This attention will undoubtedly lead to many new developments and breakthroughs in the following years, particularly in computer vision problems, as this suite of methods becomes more mature and more widely used.

## Data Availability

The data presented in this study are available upon request from the corresponding author. The data are not publicly available due to company privacy matters; however, all data contained in the dataset mentioned in the manuscript is publicly available.

## Abbreviations

The following abbreviations are used in this manuscript:

AI	Artificial Inteligence
ML	Machine Learning
DL	Deep Learning
CBIR	Content Based Image Retrieval
CNN	Convolutional Neural Network
DNN	Deep Neural Network
DCNN	Deep Convolution Neural Network
RGB	Red, Green, and Blue
